# Mental healthcare in Kenya: Exploring optimal conditions for capacity building

**DOI:** 10.4102/phcfm.v6i1.682

**Published:** 2014-10-10

**Authors:** Elijah Marangu, Natisha Sands, John Rolley, David Ndetei, Fethi Mansouri

**Affiliations:** 1 School of Nursing and Midwifery, Deakin University, Australia; 2 Africa Mental Health Foundation and University of Nairobi, Kenya; 3 Centre for Citizenship and Globalization, Deakin University, Australia

## Abstract

The global burden of disease related to mental disorders is on the increase, with the World Health Organization (WHO) estimating that over 450 million people are affected worldwide. The Mental Health Global Action Program (mhGAP) was launched by the WHO in 2002 in order to address the widening gap in access to mental healthcare in low-income countries. Despite these efforts, access to mental healthcare in low-income countries remains poor and is often described as inadequate, inefficient and inequitable, with an 85% estimated treatment gap in low-income countries, as compared with 35% to 50% in high-income countries.In this article, the authors argue that integrating mental health services into primary healthcare settings through capacity building is vital with regard to achieving mhGAP goals. The article explores the challenges to and potential enablers for the improvement of the delivery of broad-based mental healthcare services in Kenya. The authors propose the integration of the conceptual dimensions of both the cosmopolitanism and capabilities approaches as a combined strategy for dealing with capacity building in heterogeneous settings such as Kenya.

## Introduction

The World Health Organization (WHO) estimates that 450 million^1^ people worldwide experience a mental disorder, which accounts for nearly 12%^1^ of the total global burden of disease. High- and low-income countries experience a similar prevalence for mental disorders,^2,3,4^ yet large discrepancies exist between resources dedicated to mental health services in low-income countries compared with high-income countries.^2^ Kenya, a country in East Africa with a population of approximately 43 million, has less than 500 mental health professionals. Furthermore, inadequate funding and underdeveloped policy frameworks add to the challenge of delivering broad population-based mental healthcare.

This article explores the challenges facing Kenya with regard to improving the delivery of broad-based mental healthcare, including potential barriers and enablers. Both the Cosmopolitanism^5^ and Capabilities Approach^6^ as theoretical frameworks for improving mental healthcare delivery will be discussed in terms of engaging in capacity-building strategies in primary health settings.

### Mental healthcare in Kenya

Mental illness is common in Kenya, with prevalence rates of 4%^7^ for major mental disorders, which is comparable with the prevalence rates reported in high-income countries.^8,9^ Poverty, unemployment, internal conflict, displacement and HIV^7^ add to the mental health burden. Mental health services in Kenya are mainly government funded, with very few privately-funded programmes and facilities.^10^ Based in Nairobi, Mathari Hospital is the largest psychiatric hospital, providing inpatient services for the whole of Kenya.^9^

### Epidemiologic transition in Kenya and its impact on mental healthcare delivery

Omran’s^11^ theory of epidemiological transitions is useful when accounting for recent changes in disease patterns in sub-Saharan Africa and Kenya. His theory outlines three ‘ages’ of relevance to epidemiology and argues that these affect all societies. These ages are: (1) the age of pestilence and famine, characterised by high mortality rates and a life expectancy of about 30 years; (2) the age of receding pandemics and an increase in life expectancy up to 50 years; and (3) the age of degenerative diseases and fluctuating life expectancy.^11^ Regarding mental health, the burden of mental disorders is expected to increase in low- and middle-income countries such as Kenya as a result of the epidemiologic transition occurring within these countries, characterised by a marked increase in non-communicable diseases such as diabetes, cardiac disease and mental disorders.^12^ In recognition of these trends, the WHO launched the Mental Health Global Action Program (mhGAP) in 2002 in order to help address the disparity in mental healthcare between high- and low-income countries.^13^ The WHO-mhGAP package includes human resources development, increased financing and effective budgeting, advocacy, information system development and monitoring and evaluation.^13^

### Prioritising mental health in the face of other complex health issues

Since attaining independence from Britain in 1963, Kenya has engaged in a number of health reforms as a means toward strengthening health services, eradicating communicable diseases and improving health access for the whole population.^14^ Whilst there have been improvements with regard to child and maternal mortality and a reduction in morbidity and mortality rates related to communicable diseases, access to healthcare is still hampered by poverty, political instability, corruption and rapid population increase.^14,15^ Mental healthcare has received little attention in Kenyan health reform and it remains a low policy and budget priority.^10^

Prioritising resource allocation for mental health services in a country the likes of Kenya is often a challenge because of competing health priorities, including infectious diseases such as HIV, malnutrition, unsafe drinking water, malaria and increasing rates of chronic disease such as diabetes, cardiac disease and renal failure.^16^

### Policy and stigma

Mental Health Policy is integral to setting the agenda for planning and delivering mental health services.^2^ At present, Kenya has no formal, ratified mental health policy, which severely limits the mental health reform agenda.^17^ Jenkins argues that health administrators need to play a role in influencing politicians to take a more active role in advocating for mental healthcare, observing that integration of mental healthcare into the national health sector strategic plan at every level (community to tertiary) is likely to lead to better, more sustainable changes and positive health outcomes.^18^

Studies undertaken in Australia^19^ have concluded that stigmatising attitudes toward people with mental illness impact on service delivery and recovery from mental illness.^19^ Stigma and discrimination against people with mental illness (and the healthcare staff who work in mental health settings) is a significant challenge for capacity-building efforts in Kenya.^20^ and this may explain why very few health professionals choose to work in mental health services.^10^ Capacity-building efforts in Kenya must commence by, firstly, addressing the endemic issues of stigma and lack of mental health literacy in the health workforce and, secondly, addressing the society on a broader scale.^21^ Public education through social marketing is an effective way of achieving a reduction in stigma and educating the public about mental health and illness. The success of social marketing approaches in addressing HIV prevention and public education in Kenya, Ghana and Uganda^22^ confirms the power of social marketing for health promotion and capacity-building; these same approaches could be applied to mental health capacity building.

### The limitations of a traditional mental health workforce

A major challenge for mental healthcare in Kenya is the severe shortage or, in some regions, total lack of a specialist mental health workforce.^10^ The WHO^1^ reports the global median mental health workforce to population ratio as 10.7 staff per 100 000, which is in stark contrast to current resourcing to mental health in Africa, reported as being 1.7 mental health staff per 100 000. Kenya has 54 psychiatrists, 418 trained psychiatric nurses, 10 medical social workers and very few psychologists^10^ to cater to a population of about 43 million,^23^ 4%^7^ of whom are likely to suffer from a major mental disorder. The low levels of mental health literacy amongst health workers at all levels of the Kenyan healthcare system compounds the problem of lack of access to mental healthcare resulting from mental health workforce shortages.^20^

Compounding the workforce issues, a ‘brain drain’, or the trend of losing trained health workers from low- to high-income countries has had a significant impact on healthcare in sub-Saharan Africa.^24^ Of the 74 psychiatrists who have been trained in Kenya since 1980, only 54 have remained to practise in Kenya.^25^ There are 418 trained psychiatric nurses in Kenya, but only 250 are currently working in mental health services; others have either emigrated or are working in non-governmental organisations.^10^ Attracting and retaining a specialist mental health workforce remains a significant challenge in Kenya.^9^

### The problem

Kenya faces a set of complex challenges if it is to realise its goal of improved mental healthcare. The devolving healthcare system away from a Nairobi-centric model to the Counties^26^ is an important indicator. However, the persisting lack of investment in real terms, insistence on maintaining traditional approaches in training a professional mental health workforce and the pervading stigma of mental illness continue to hamper the best of intentions.^20^

Improving mental healthcare delivery for the broadest base of the population will require a different approach from that taken in the past. Central to the WHO’s mhGAP initiative^13^ is for countries to have a robust and efficient mental health workforce at the primary healthcare level.^27^ Thornicroft and Tansella, similarly, recommend low-income countries to focus resources on primary healthcare with specialist back-up and the gradual increase of specialist mental health component services as more resources become available.^28^ Thornicroft and Tansella’s model can be achieved in Kenya by training and by the task shifting of basic mental health assessment, diagnostic and treatment functions from tertiary level institutions to primary healthcare settings through education, training and other knowledge dissemination strategies. In this approach, the existing network of public and non-governmental organisations, including faith-based health facilities at the primary healthcare level, would be used as a vital component of the educational capacity-building strategy. Adopting a primary healthcare focus optimises the potential for a more sustainable and broad-based reach to basic mental healthcare in Kenya.

## Theoretical approaches

Two theoretical frameworks are relevant with regard to guiding capacity building: Cosmopolitanism and the Capabilities Approach. The former provides a philosophical foundation that locates the Kenyan context within a broader humanitarian imperative,^6^ whilst the Capabilities Approach, an economic model for developing capacity, would provide an operational framework^29^ consistent with the goals of Cosmopolitanism.

### Cosmopolitanism: Understanding the Kenyan historical, social and political context

Whilst cosmopolitanism, as a philosophical construct, has been around for centuries and has often indicated an outward orientation, along with a capacity to exhibit care and empathy toward strangers and the less-fortunate members of society, some scholars, including Delanty^30^ suggest that cosmopolitanism can best be represented as a ‘transformative condition’ concerned with possibilities in the present. It is a ‘condition’ of openness to the world that involves social transformation in light of encounters with the ‘other’.^30^ As such, cosmopolitanism has been applied successfully to studies involving complex political, economic and social settings.^5,31^ Kenya has been described similarly as a multi-cultural and multi-linguistic society blending African, Arabic and European elements.^32^ Strand (2010) suggests that cosmopolitanism is a conceptual approach rather than a prescriptive theoretical framework; it is a way of seeing the world, on the one hand, as an evolving and complex social reality and, on the other, as a paradigm of political and social analysis;^33^ as such, is an appropriate lens through which to understand and then analyse the Kenyan health context. Kenya is a post-colonial, emerging economy, with many political and social challenges that impact on health resourcing and planning. With multiple government and non-governmental organisations involved in healthcare delivery, as well as having a rich cultural heritage and diverse ethnic population, Kenya is ‘cosmopolitan’ in the truest sense of the word. Cosmopolitanism, in this context, capitalises on these unique contextual factors with regard to both planning and delivering capacity-building strategies and provides a standpoint from which to examine ‘who’ the actors are and ‘what’ their agendas may be;^33^ this information is critical with regard to planning effective and sustainable mental healthcare capacity-building strategies for Kenya.

### Capabilities Approach: Mental health capacity building in Kenya

Sen’s Capabilities Approach (CA)^34^ is a prominent framework that could be employed for the design and development of a mental health capacity-building model for Kenya. The CA is essentially an economic model that challenges the welfare approach and considers the different *Commodities* and *Capabilities* within the society that can enable its *Abilities* (development). Kenya is currently in a state of political stability, giving rise to positive developments in a number of sectors, including health.^35^ According to the current (2013) World Bank forecast,^35^ the Kenyan economy is set to grow by at least 6% in the next few years and inflation will be less than 10%.^35^ Using Sen’s approach, an appraisal of the current strengths in the Kenyan economy and healthcare system can be achieved; this is an important step in developing mental health programmes that are sustainable into the future, with less dependence on foreign funding. Political stability and economic prosperity in Kenya are factors that optimise the likelihood of reform in the health sector,^24^ including mental healthcare.

## Building primary healthcare capabilities

Despite many political, economic and social challenges that have impeded mental health services improvements and reform, Kenya has many strengths that can be harnessed in order to enable further development of its mental healthcare system.

### The stakeholders

Kenya has a wide network of non-governmental organisations, charitable organisations and development partners such as the United Nations International Development Agency (USAID).^26^ These stakeholders have been vital to healthcare delivery in Kenya for many years^32^ and in involving them in collaborative mental health capacity building is likely to add significant value to strategies developed by the Kenyan Ministry of Health. The African Mental Health Foundation and Basic Needs (UK) are examples of stakeholders currently involved in advocacy, planning and delivery of mental healthcare in Kenya.^8^ The activities of these groups include engagement with mental health consumers, public education, engaging in research on psychotrauma which has been common during times of political instability and offering mental health outreach services in rural and remote areas of Kenya.^7^ Strengthening these existing collaborative partnerships through educational exchange, training, pooling of resources and collaborative research would be a logical way forward when planning future mental health reform and capacity building in Kenya.

### Mental health literacy

Jorm et al. define mental health literacy as ‘knowledge and beliefs about mental disorders which aid their recognition, management and prevention’.^36^ Mental health literacy is important for both the general public and health professionals for the early identification, treatment of and ongoing support for people with mental disorders.^37^ The ability to read and write is key with regard to getting information about mental disorders to the public or primary healthcare workers. Literacy levels in Kenya are estimated to be over 85%^35^ and there has been an increase in the number of higher education institutions in recent years.^15^ The high levels of literacy and increasing availability of educational opportunities in Kenya are positive attributes that will present opportunities for capacity building through mental health literacy. Incorporating mental health literacy programmes^38^ into education programmes in schools and universities and through social marketing-based mental health promotion campaigns, will be an important step in increasing awareness of mental disorders and fighting the associated stigma.

### Traditional and faith healers

Kenya has a parallel healthcare system, which works alongside the Western-based system and includes indigenous ‘traditional’ and faith healers;^3^ yet the role of traditional healers in the Kenyan healthcare system is neither officially recognised nor remunerated.^9^ A study undertaken amongst people with mental illness in Nairobi by Ndetei et al.^39^ found that the majority of patients who participated in the study consulted both Western medical practitioners as well as traditional and faith healers; the authors thus concluded that there was a need for linkages between Western medical practitioners and traditional healers because their roles were complementary.^39^ Traditional and faith healers are increasingly involved in delivery of healthcare in rural and remote settings in Kenya; examples include traditional birth attendants and tuberculosis programmes.^14^ Mental health capacity-building initiatives implemented in Kenya should include faith and traditional healers. An important first step would be to recognise their role and then to offer training in basic mental healthcare and to clarify referral pathways for the more difficult cases that they are not able to deal with. This approach would acknowledge the cultural importance^40^ of this dimension of healthcare in Kenya and recognise the capacity-building potential of strengthening and working collaboratively with existing healthcare workers with formal medical or traditional backgrounds.^39^ This inclusive approach is also consistent with the core values of cosmopolitanism.

### Technology and capacity building

In recent years there has been a marked increase in the use of mobile technologies in Kenya; and it is estimated that up to 70%^23^ of the adult population in Kenya use their mobile phone for mobile banking.^23^ Similarly, mobile phones in Kenya are now increasingly used by farmers, pastoralists and business people in order to monitor market conditions.^41^ Trials using mobile technology to improve antenatal care and tuberculosis programmes in Kenya have shown positive results.^26^ The increasing use of technology in Kenya presents opportunities to use this technology for mental healthcare capacity-building strategies, for example, in training health workers in rural and remote areas and as a potential platform for public mental health promotion through a variety of social networking platforms accessible via phones and other mobile technologies. Mobile technologies are also useful with regard to extending the reach of clinical care, for example, to provide follow-up for consumers with mental illness and to enhance monitoring and communication about mental disorders within and across health facilities.

### Devolution

In the past, Kenya has had a highly-centralised health system whereby the vast majority of health resources were concentrated in the capital city, Nairobi,^32^ which made access to healthcare difficult for people living in rural and remote areas. The recent devolution and decentralisation of government functions to the county level^35^ has potential to lead to more equitable use of available health resources if implemented. There is already evidence emerging^35,42^ to suggest that counties within Kenya are reassessing the needs of their jurisdictions and planning service development targeting areas of healthcare neglected in the previous system;^35^ this, in turn, could present opportunities for mental health capacity building in the primary health sector.

## Conclusion

Whilst Kenya is not unique in the prevalence of mental illness, it does, as with other low-income countries, experience an overwhelming gap in basic mental healthcare delivery to the majority of the population. The growing political stability and burgeoning economic prosperity bode well for the improvement of healthcare in general, yet substantial investment in building capacity for effective mental healthcare is still vital. Whilst, philosophically, cosmopolitanism is not a new idea, its application in the context of improving mental healthcare in Kenya is innovative. The transformative nature of cosmopolitanism with its emphasis on the possibilities of the present can aid in developing sustainable solutions that recognise the critical importance of local stakeholders and which capitalise on Kenya’s unique cultural context.

The other deeply complementary framework discussed in this article, namely, the Capabilities Approach, looks to the strengths inherent in the context from which to build capacity for better mental healthcare. These strengths include existing literacy levels, the level of population connectivity through technology and alternative healthcare models such as traditional healers who can complement the efforts of Western-trained healthcare workers. In keeping with WHO recommendations, focusing on capacity building at the primary healthcare level may provide a sustainable way of improving access to basic mental healthcare.
